# Preparation of Polyacrylonitrile-Based Immobilized Copper-Ion Affinity Membranes for Protein Adsorption

**DOI:** 10.3390/membranes13030271

**Published:** 2023-02-24

**Authors:** Yin-Jie Yang, Hou-Chien Chang, Min-Ying Wang, Shing-Yi Suen

**Affiliations:** 1 Department of Chemical Engineering, National Chung Hsing University, Taichung 402, Taiwan; 2 Graduate Institute of Biotechnology, National Chung Hsing University, Taichung 402, Taiwan; 3 i-Center for Advanced Science and Technology, National Chung Hsing University, Taichung 402, Taiwan

**Keywords:** polyacrylonitrile membrane, immobilized metal-ion affinity membrane, protein adsorption, adsorption arrangement

## Abstract

A polyacrylonitrile (PAN)-based immobilized metal-ion affinity membrane (IMAM) was prepared with a high capacity for protein adsorption. PAN was selected as the substrate due to its excellent thermal and chemical stability. The cyano groups on the PAN membrane were substituted with carboxyl groups, followed by reactions with ethylenediamine (EDA) and ethylene glycol diglycidyl ether (EGDGE) to produce the terminal epoxy groups. The chelating agent iminodiacetic acid (IDA) was then bound to the modified PAN membrane and further chelated with copper ions. The immobilized copper ion amount of membrane was analyzed to obtain the optimal reaction conditions, which were 60 °C/3 h for EDA coupling and 60 °C/4 h for EGDGE grafting. Furthermore, under the use of minor IDA and copper ion concentrations, the immobilized copper ion capacity of the IMAM was 4.8 μmol/cm^2^ (253.4 µmol/mL, or 1.47 μmol/mg). At a neutral pH, the cationic lysozyme exhibited a large adsorption capacity with the IMAM (1.96 μmol/mL), which was most likely multilayer binding, whereas the adsorption capacity for bovine serum albumin (BSA) and histidine-tagged green fluorescent protein (GFP-His_6_) was 0.053 μmol/mL and 0.135 μmol/mL, respectively, with a monolayer adsorption arrangement. The protein desorption efficiency was greater than 95%, implying that the prepared IMAM could be reused for protein adsorption.

## 1. Introduction

Bioseparation and purification are popular and vital technologies in biochemical, food, pharmaceutical, medical and other related industries [1,2]. In particular, effective methods of target biomolecule purification are required for rapid progress in biotechnology [3]. Bioseparation and purification methods are usually designed from discrepancies in biomolecule properties such as molecular size (size exclusion), charge type (ion exchange), hydrophobicity (hydrophobic interaction) or specific affinity [4,5,6,7,8].

Among the specific affinity methods for isolating target biomolecules, immobilized metal-ion affinity chromatography (IMAC) is a popular technique. It utilizes the chelators coupled on solid particles to immobilize the metal ions (as electron-pair acceptors), which could specifically bind with the exposed electron-donating amino acid residues (e.g., histidine, cysteine, tryptophan, etc.) on biomolecule surfaces [1,2,3,8,9,10,11,12,13]. The metal ions immobilized on a solid carrier are usually first-row transition metals (Cu^2+^, Ni^2+^, Zn^2+^, Co^2+^, etc.) [5,9,13,14,15,16]. The vacant valence orbitals of a first-row transition metal provide excellent stability for the coordination compound formed. Biomolecules containing specific amino acids are absorbed into the metal ion-immobilized solid carrier with specific affinity, and thus separation and purification can be carried out [1,2,3]. This method offers the advantages of high capacity, high recovery, complete regeneration and low cost [12,16].

However, process time and cost are major concerns for practical chromatographic processes [4,6,12]. Using big, porous particles as solid carriers usually comes with a prolonged diffusion time in torturous pores. On the other hand, the solid particle’s packing will be tight as the particle size decreases, which demands greater pressure to maintain a suitable fluid velocity. The higher energy consumption thus constitutes a main disadvantage of such a separation process.

Via replacing the chromatographic solid particles with film-type membranes, better features and performance could be achieved for so-called membrane chromatography or the adsorptive membrane process: simple design, low pressure drop, fast mass transfer, small process time and easy scale-up [3,4,5,6,12,14,15,16,17,18,19]. The membrane is proposed to take the position of solid particles, with the advantages of allowing solutes to enter the macropores of a thin film through convection; such an adsorption process could thus be conducted under lower pressure and with quicker mass transport, leading to lower energy consumption and less cost. Moreover, the adsorptive membrane could offer a benefit of a simpler scale-up by means of stacking several flat membranes together, winding a large flat membrane spirally or gathering a bundle of tubular membranes [4,5,6,17,18].

The efficiency of affinity adsorption depends on the adsorption capacity, stability and features of the solid adsorbents. Accordingly, the matrix material, spacer arm, chelating agent and the immobilized metal ions of the IMAC or immobilized metal-ion affinity membrane (IMAM) will greatly affect the adsorption effectiveness. As reported in the literature [3,5,12,13,14,15,20,21,22,23,24], the polymeric matrices for IMAMs include cellulose, polyamide, polyvinylbutyral, polysulfone, poly(vinyl alcohol-co-ethylene) and so on. Hydrophilic polymers are more commonly selected; in addition, the polymer functionality for further ligand coupling is also important. In this study, porous polyacrylonitrile (PAN) membrane with reactive cyano groups was employed as the substrate of IMAM. Due to its excellent thermal stability, resistance to most solvents, antibacterial property, weather resistance, commercial availability and low cost, the application of the PAN membrane has attracted a lot of attention in recent research [25,26,27,28,29,30]. However, its hydrophobic nature has to be modified through introducing the hydrophilic groups for application in biomolecule adsorption [30]. The substitution of a cyano group by a hydroxyl group, amino group, sulfhydryl group or carboxyl group is usually carried out [31].

Prior to metal ion immobilization, the chelating agent needs to be bound to the membrane matrix. The common choices of chelating agents are multidentates, such as iminodiacetic acid, tridentate (IDA), nitrilotriacetic acid, tetradentate (NTA), and N,N,N-tris(carboxymethyl)ethylenediamine, pentadentate (TED) [1,5,13,14,16,32,33], as well as triazine dyes [3,5,34,35,36]. Nevertheless, the direct coupling of a chelating agent onto a polymeric membrane matrix is not usually easy. A spacer-arm molecule with two terminal reactive groups is thus utilized. Another function of the spacer arm is to lengthen the distance between the immobilized metal ions and the solid substrate for assisting the big biomolecule to contact the metal ions. Common selections of the spacer arm are diamine, diol, epichlorohydrin, etc. [1,14,15,21,23]. Among the multidentates, tridentate may not afford a stronger immobilization with metal ions; on the contrary, its related adsorption strength with biomolecules is higher [5]. A better chelating efficiency could be achieved by using IDA and copper ions, according to the literature [34]. The practical applications of IMAM reported in the literature are broad, such as the purification of commercial human serum albumin (HSA) solution [3], purification of bovine liver catalase [3], isolation of lysozyme from egg white [12,37], immunoassay diagnostics [13], purification of the VP3 protein of infectious bursal disease [14], simultaneous purification and immobilization of scaffolding protein genes [15] and purification of a histidine-tagged recombinant protein from *Escherichia coli* [38], etc.

In the present study, the cyano groups of the PAN membrane were substituted for carboxyl groups first, followed by two grafting reactions of diamine and diepoxide spacer arms, then the coupling of chelator IDA. Finally, copper ions were chelated with IDA via the use of copper (II) sulfate. Based on the capacity of immobilized copper ions, the optimal conditions for preparing the PAN-based IMAM were determined. This paper investigates the protein adsorption behaviors for the prepared IMAM. The tested proteins included two model proteins, lysozyme and bovine serum albumin (BSA), and histidine-tagged green fluorescent protein (GFP-His_6_). At a neutral pH, the lysozyme is cationic and BSA is anionic. Nonspecific and specific bindings were analyzed from the results of their adsorption onto the membranes obtained at each modification step in the process of IMAM preparation. Possible adsorption arrangements on IMAM for these three protein molecules were also interpreted, which will be beneficial for exploring the IMAM application in this field.

## 2. Materials and Methods

### 2.1. Materials

**Figure 1:**
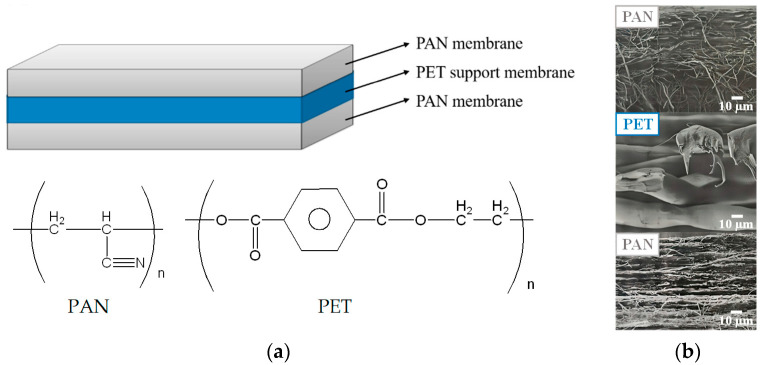
(**a**) Structure of the 3-layer PAN membrane used in this work and (**b**) cross-section SEM images for each layer.

The 3-layer PAN membrane (each layer has a thickness of 95 μm), whose structure and cross-section SEM images are shown in Figure 1, was a kind gift from the Taiwan Textile Research Institute (New Taipei City, Taiwan). The reagents and chelating agent adopted for the preparation of IMAM included: ethylenediamine (EDA) from Tedia (Fairfield, OH, USA), ethylene glycol diglycidyl ether (EGDGE) from TCI (Tokyo, Japan) and iminodiacetic acid (IDA) from Acros (Morris Plains, NJ, USA). The source of copper ions was copper (II) sulfate pentahydrate (Showa, Gyoda, Japan). The model proteins investigated in this work were lysozyme (MW = 14,300, pI = 11) and bovine serum albumin (BSA, MW = 69,293, pI = 4.7), both purchased from Sigma (Taufkirchen, Germany). The histidine-tagged green fluorescent protein GFP-His_6_ (MW = 28,400, pI = 6) was prepared in Prof. Min-Ying Wang’s lab at NCHU, Taichung, Taiwan. The solvents such as NaOH (Showa, Gyoda, Japan), HCl (SCHARLAU, Barcelona, Spain), Tris (Bio Basic, Markham, ON, Canada) and toluene (ECHO, Toufen, Miaoli, Taiwan) was used as received.

### 2.2. Preparation of IMAM

**Figure 2:**
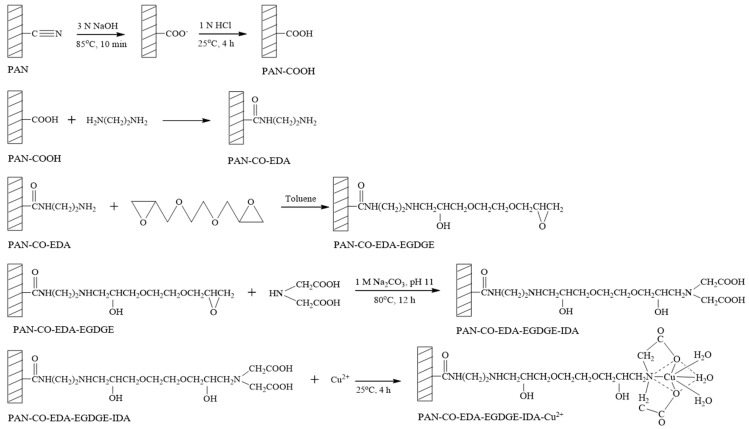
Modification steps of PAN membrane for the preparation of IMAM.

For the preparation of IMAM, the modification steps of the PAN membrane are shown in Figure 2. In the first step, one piece of 25 mm PAN membrane disc (16 mg/piece) was reacted with 30 mL of 3 N NaOH solution at 85 °C for 10 min, followed by immersing in 1 N HCl solution at room temperature for 4 h. The cyano groups of PAN membrane were replaced with carboxyl groups as PAN-COOH. In the second step, the PAN-COOH membrane was placed in 10 mL of 7.5 M EDA solution in a variety of temperatures and times in order to form PAN-CO-EDA. The membrane was then rinsed with deionized water and dried. In the third step, the PAN-CO-EDA membrane was immersed in 10 mL of 3.2 M EGDGE solution (in toluene) at various temperatures and times to graft the EGDGE onto the membrane (PAN-CO-EDA-EGDGE) and produce the terminal epoxy groups. The membrane was dried in a vacuum oven for 4 h to remove the solvent. In the fourth step, the membrane was submerged in 10 mL of 0.2 M or 1 M IDA solution containing 1 M Na_2_CO_3_ (pH 11) at 80 °C for 12 h to fix the chelating agent (IDA) on the membrane (PAN-CO-EDA-EGDGE-IDA). The final step was to chelate the copper ions onto the membrane (PAN-CO-EDA-EGDGE-IDA-Cu^2+^) by submersing the membrane in 5 mL of 0.05 M or 0.5 M copper (II) sulfate solution at room temperature for 4 h. Next, the membrane was washed with deionized water and 50 mM Tris buffer (pH 7) to release the unstably bound copper ions.

### 2.3. Membrane Characterization

ATR-FTIR (FTIR-4100, JASCO, Tokyo, Japan) was adopted to characterize the functional groups of membrane after each modification step. The morphology of the membrane surface was detected by SEM (JSM-6700F, Jeol, Tokyo, Japan).

The ion-exchange capacity (IEC) of the membranes was measured as follows: one piece of 25 mm membrane disc was submersed in 20 mL of 0.1 N HCl solution in a shaker at room temperature for 24 h. The membrane was then rinsed with deionized water several times to eliminate the acid trace. Next, the membrane was equilibrated with 0.01 N NaOH solution with shaking at room temperature for 24 h. After that, the alkalinity reduction in the remaining NaOH solution was measured by titration, using 0.01 N HCl. The IEC of membrane was calculated by the following equation:

(1) $$\mathrm{IEC}\;(\mu \mathrm{mol}/{\mathrm{cm}}^{2})=\frac{{(N}_{\mathrm{NaOH},0}-N_{\mathrm{NaOH},E}{)V}_{\mathrm{NaOH}}}{A}$$

where N_NaOH,0_ and N_NaOH,E_ represent the molar concentrations of NaOH solution before and after the equilibration, respectively. V_NaOH_ is the volume of NaOH solution. A is the membrane area.

Two instruments were employed to determine the concentration of copper ions in copper (II) sulfate solution: UV-Vis (UV-1601, Shimadzu, Sydney, Australia) at 800 nm and ICP-AES (ICAP9000, Jarrell-Ash, Franklin, MA, USA). The amount of immobilized copper ions on the membrane was evaluated as follows:

(2) $${\mathrm{Cu}}^{2+}\;\mathrm{capacity}\;(\mu \mathrm{mol}/{\mathrm{cm}}^{2})=\frac{{(C}_{\mathrm{Cu},0}-C_{\mathrm{Cu}}{)V}_{\mathrm{Cu}}}{A}$$

where C_Cu,0_ and C_Cu_ are the concentrations of copper ions in the solution before and after the final chelation step, respectively. V_Cu_ is the volume of copper (II) sulfate solution.

### 2.4. Batch Protein Adsorption and Desorption Experiments

The tested proteins were lysozyme, BSA and GFP-His_6_. The buffer used to prepare the protein solution was 50 mM Tris, pH 7 for lysozyme and BSA, as well as 20 mM Tris, pH 8, for GFP-His_6_. One piece of 25 mm membrane disc was incubated in 5 mL of a protein solution of a certain initial concentration for 24 h at room temperature. The lysozyme or BSA concentration in solution was measured via UV-Vis at 280 nm, whereas the GFP-His_6_ concentration in solution was analyzed by detecting its fluorescence intensity with a multimode microplate reader (Infinite m200 Pro, Tecan, Männedorf, Switzerland) at an excitation wavelength of 488 nm and an emission wavelength of 518 nm. The adsorbed protein amount (q) was determined by the following equation:

(3) $$q\;(\mu g/\mathrm{mL}\;\mathrm{or}\;\mu \mathrm{mol}/\mathrm{mL})=\frac{{(C}_{\mathrm{protein},0}-C_{\mathrm{protein}})V_{\mathrm{protein}}}{V_{\mathrm{mem}}}$$

where C_protein,0_ and C_protein_ represent the protein concentrations in solution before and after the batch adsorption experiment, respectively. V_protein_ is the protein solution volume. V_mem_ is the membrane volume, 0.093 mL, where only the thickness of 2 PAN layers was considered, since the PET layer was not modified and did not have the protein adsorption capability. In addition, a laser scanning confocal microscope (LSCM, FV1000, Olympus, Tokyo, Japan) was adopted to observe the fluorescence image for the GFP-His_6_-adsorbed IMAM.

After the batch adsorption under an initial concentration of 0.5 mg/mL, the IMAM with adsorbed protein was immersed in 5 mL of elution solution for 2 h at room temperature. The elution solution was 250 mM imidazole in 50 mM Tris, pH 7. The protein concentration in the elution solution was also measured spectrophotometrically using an appropriate calibration curve. The desorbed protein amount was then calculated by multiplying its concentration in the elution solution with the solution volume.

## 3. Results and Discussion

### 3.1. IMAM Characterization

**Figure 3:**
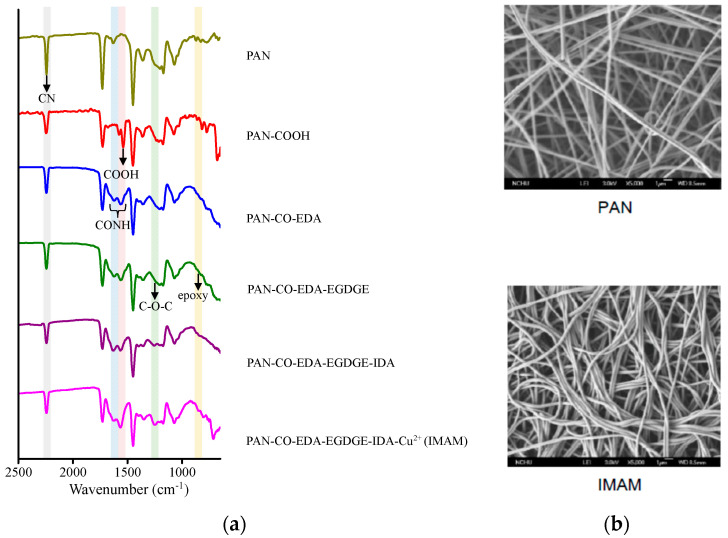
(**a**) ATR-FTIR spectra on various membrane modification steps in the process of IMAM preparation and (**b**) SEM images of the pristine PAN membrane and IMAM.

Figure 3a shows the related ATR-FTIR spectra on various membrane-modification steps in the process of IMAM preparation. Significant differences with the spectra can be observed within the wavenumber range of 650–2500 cm^−1^. The main functional group on the pristine PAN membrane was the CN group at 2240 cm^−1^ [26]. After replacing the CN groups with the COOH groups, the COOH peak on the PAN-COOH membrane was detected at 1570 cm^−1^ [26], validating the successful modification. However, the CN peak did not totally disappear, which is probably due to an incomplete reaction of CN into COOH. Via the IEC measurement, the number of COOH groups on the PAN-COOH membrane was quantified as 27 μmol/cm^2^ frontal membrane area. In the second modification step with EDA, the CONH group was created and presented at 1550–1640 cm^−1^ [39] in the spectrum of PAN-CO-EDA. The next step was to graft EGDGE onto the membrane for the formation of terminal epoxy groups. As indicated in the literature using poly(EGDGE) for modification, the characteristic peak of epoxy group (oxirane ring vibration) was exhibited at 800 or 865 cm^−1^ [40,41] and that of C-O-C (stretching vibration) in the EGDGE structure at 1126 cm^−1^ [40]. In Figure 3a, these two characteristic peaks of EGDGE were very tiny in the spectrum of PAN-CO-EDA-EGDGE, while they were more evident (840–870 cm^−1^ and 1250 cm^−1^) for the subsequent PAN-CO-EDA-EGDGE -IDA and PAN-CO-EDA-EGDGE-IDA-Cu^2+^ membranes. In the fourth stage, the chelator IDA reacted with the epoxy groups of PAN-CO-EDA-EGDGE. After this stage, the color of the membrane changed from white to light yellow, confirming the successful IDA reaction. In the final stage, the copper ions were immobilized onto the membrane and the membrane color became blue-green. No new distinctive peaks appeared at the last two spectral curves in Figure 3a, since the copper ions could not be detected with ATR-FTIR.

The membrane morphology was observed by SEM (5000-fold magnification) and the images of pristine PAN membrane and IMAM are displayed in Figure 3b. As shown in the SEM images, the pristine PAN membrane contained long fibers and it was not damaged under the reaction conditions of strong acid, strong base and high temperature. This result verifies again that our PAN-based IMAM preparation process was satisfactory.

A variety of reaction time and temperature for EDA and EGDGE reactions were tested in this work. Based on the use of 0.2 M IDA and 0.05 M CuSO_4_ in the subsequent reactions, the amount of immobilized copper ions on the resultant IMAM determined via UV-Vis at 800 nm is listed in Table 1. The highest copper ion capacity of 4.8 ± 0.08 µmol/cm^2^ was obtained under the EDA reaction condition at 60 °C, 3 h and EGDGE reaction at 60 °C, 4 h. The amount of immobilized copper ions in this optimal condition was also measured through ICP-AES, and the result was 4.8 ± 0.09 µmol/cm^2^. Both values were almost identical. In comparison with the number of active COOH groups on the PAN-COOH membrane (27 μmol/cm^2^), about 18% of the COOH groups were utilized and modified into the specific binding sites, i.e., immobilized copper ions. However, it should be noted that this immobilized copper ion capacity (4.8 µmol/cm^2^, 253.4 µmol/mL or 1.47 μmol/mg), under the condition of lower chelator or copper ion concentrations, was 4–250-fold greater than those reported in the literature (0.17 µmol/cm^2^ [23], 0.42–0.53 µmol/cm^2^ [42], 1.22 µmol/cm^2^ [34] and (5.82 ± 0.22) × 10^−3^ µmol/mg [38]).

To further improve the immobilized copper ion capacity, the concentrations of the IDA and copper (II) sulfate were increased to 1 M and 0.5 M, respectively, while both the EDA and EGDGE reactions were set at the optimal condition. The resulted immobilized copper ion amount was 5.2 ± 0.7 µmol/cm^2^ via UV-Vis and 5.1 ± 0.1 µmol/cm^2^ via ICP-AES. Both values were very close. Since the increase in IDA and copper (II) sulfate concentration could only enhance the immobilized copper ion capacity at 6–8%, lower reagent concentrations of 0.2 M IDA and 0.05 M CuSO_4_ were recommended for greener IMAM preparation. The IMAM prepared using the optimal and greener conditions was accordingly employed in the follow-on adsorption investigation.

### 3.2. Protein Adsorption onto IMAM

#### 3.2.1. Nonspecific Binding and Specific Binding

The interactions between protein and the immobilized metal ion affinity adsorbents (IMAC or IMAM) could be categorized into [4,5,12]: (1) affinity binding between the specific amino acid (ex. histidine) of protein and the immobilized metal ions, (2) electrostatic attraction between protein and the immobilized metal ions, (3) electrostatic attraction between protein and the charged groups of the adsorbent and (4) hydrophobic interaction between the nonpolar moiety of the protein and hydrophobic groups of the adsorbent. The latter two interactions belong to nonspecific binding [5,32]. In the case of the reaction being incomplete during the IMAM preparation process, the functional groups left on the membrane surface may cause nonspecific binding with the target proteins. In this study, lysozyme and BSA were selected as the target proteins for specific and nonspecific binding tests. The protein solution was prepared in Tris buffer, pH 7. At this neutral pH, the lysozyme is positively charged since it has the pI of 11, whereas BSA with a pI of 4.7 is negatively charged. Thus, their electrostatic bindings with the adsorbent represent the cationic interaction and anionic interaction, respectively.

**Figure 4:**
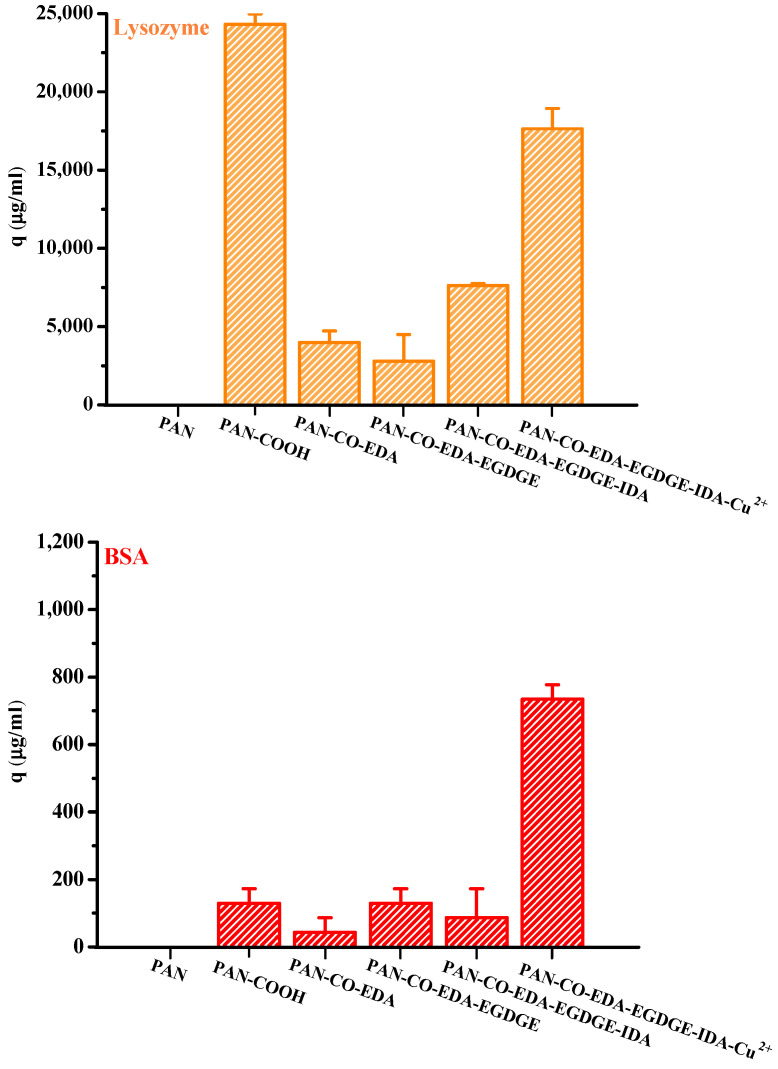
Protein adsorption results for the membranes obtained at each modification step in the process of IMAM preparation (under optimal reaction conditions) using the initial concentration of 0.5 mg/mL at room temperature for 24 h.

Figure 4 presents the lysozyme and BSA adsorption results for the membranes obtained at each modification step in the process of IMAM preparation (under optimal reaction conditions) using the initial concentration of 0.5 mg/mL at room temperature for 24 h. It could be witnessed that the pristine PAN membrane did not have any protein adsorption capability. After the first modification step where the end COOH groups were created on the membrane, lysozymes exhibited a very high adsorption amount and BSA had a small binding quantity. Since the solution pH (7) was higher than its pK_a_, the COOH groups of PAN-COOH membrane were dissociated into negatively charged carboxylate ions [26]. Consequently, cationic lysozyme molecules could be easily bound with these carboxylate ions through electrostatic force, belonging to the nonspecific binding in the case (3) mentioned above. Although the repulsion between anionic BSA and COO^−^ should have inhibited BSA adsorption onto the PAN-COOH membrane, some local positively charged moieties or hydrophobic moieties on the BSA molecule may still bind with the COO^−^ groups or hydrophobic groups of membrane, resulting in minor adsorption capacity (nonspecific binding of case (3) or case (4)).

As for the membranes attained in the second and third steps during the IMAM preparation process (PAN-CO-EDA and PAN-CO-EDA-EGDGE), their nonspecific protein adsorption quantities were low, Figure 4. By the lack of the possible bindings between the protein and the terminal amine or epoxy groups of membrane, the inapparent adsorption amounts may be accounted for the binding with the residual COO^−^ groups from the incomplete membrane modification or hydrophobic interaction. After the fourth modification stage combining IDA onto the membrane, the nonspecific adsorption of the lysozyme on PAN-CO-EDA-EGDGE-IDA increased via electrostatic attraction with the COO^−^ groups of IDA. Simultaneously, the BSA-binding capacity was reduced at this stage, owing to the repulsion force. The lysozyme adsorption quantity at this stage was not as high as that for PAN-COOH, which may be attributed to the steric hindrance between the big protein molecule and the long spacer arm.

In the final modification step, the copper ions were successfully immobilized onto the PAN membrane (PAN-CO-EDA-EGDGE-IDA-Cu^2+^) as the IMAM. Both the adsorption effects of the lysozyme and BSA with the IMAM were significant, as presented in Figure 4. The specific binding of protein on the PAN-based IMAM was much more than the nonspecific bindings for the membranes obtained at the intermediate modification steps (PAN-CO-EDA, PAN-CO-EDA-EGDGE, and PAN-CO-EDA-EGDGE-IDA), implying that our IMAM is an effective tool for protein adsorption.

To further understand the efficiency of protein recovery from this IMAM, the desorption process was conducted via immersing the IMAM with adsorbed proteins in a simple elution solution (adding the displacement agent, imidazole, in loading buffer) for 2 h at room temperature. This is a ligand-exchange method [5]. By comparing it to the adsorbed quantity, the desorption percentage was about 97% for the lysozyme and 95% for BSA, similar to the desorption efficiency using 0.5 M NaCl and 500 mM imidazole as the elution media in the literature [12]. The use of a medium imidazole concentration (250 mM) alone could effectively elute the adsorbed proteins from the prepared PAN-based IMAM. A harsher elution condition (such as a higher imidazole concentration, the addition of salt, or the use of stronger metal-chelating agent, e.g., EDTA) is not necessary.

**Figure 5:**
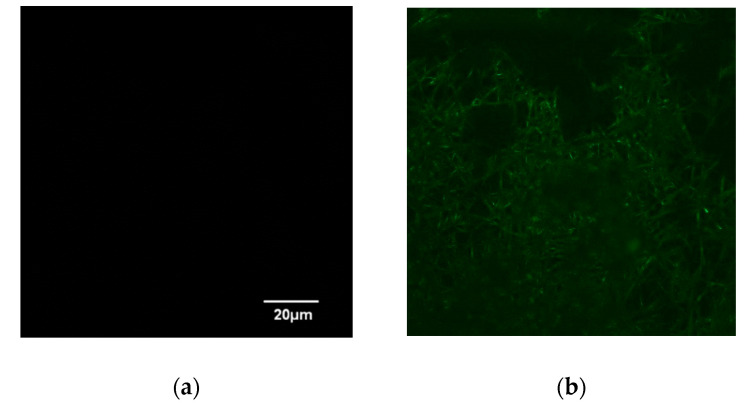
LSCM images of (**a**) IMAM and (**b**) GFP-His_6_-adsorbed IMAM.

This study also examined the specific binding of a histidine-tagged protein, GFP-His_6_, with the prepared IMAM. The adsorption pH was set at pH 8. After adsorption for 24 h to achieve the adsorption equilibrium, LSCM was adopted to observe the fluorescence image for the GFP-His_6_-adsorbed IMAM. As displayed in Figure 5, no fluorescence was detected for the prepared IMAM. After the adsorption of GFP-His_6_ onto the IMAM, the green color was evenly distributed on the whole IMAM, indicating successful specific binding.

#### 3.2.2. Adsorption Isotherm and Protein Adsorption Arrangement

**Figure 6:**
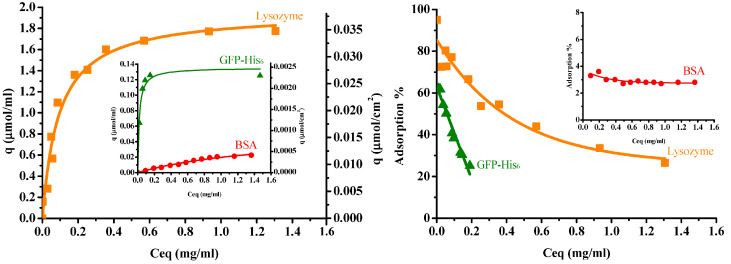
Protein adsorption isotherms (**left**) and adsorption percentages (**right**) for the prepared PAN-based IMAM at room temperature (pH 7 for lysozymes and BSA, pH 8 for GFP-His_6_).

Figure 6 illustrates the protein adsorption isotherms of three single proteins for the IMAM at room temperature (pH 7 for lysozyme and BSA, pH 8 for GFP-His_6_), along with their adsorption percentages. When adsorbed onto the IMAM, the protein molecules could form either a monolayer or multilayers. As observed from the experimental data of adsorption isotherms in Figure 6, the monolayer formation seems to occur most likely in all the three protein cases. The isotherm data were thus fitted with the Langmuir equation, a popular model for monolayer adsorption:

(4) $$\;q=\frac{q_{m}C_{\mathrm{eq}}}{K_{d}{+C}_{\mathrm{eq}}}$$

where q_m_ is the maximum adsorption capacity (μmol/mL), C_eq_ is the equilibrium concentration in solution (mg/mL), and K_d_ is the equilibrium dissociation constant (mg/mL). The fitting results are presented in Figure 6 as smooth curves. As shown in Figure 6, the isotherm data were in good agreement with the fitting results. The fitted q_m_ values were: 1.96 ± 0.082 μmol/mL for lysozyme (0.037 μmol/cm^2^ or 0.0114 μmol/mg), 0.053 ± 0.008 μmol/mL for BSA (0.001 μmol/cm^2^ or 0.00031 μmol/mg), and 0.135 ± 0.006 μmol/mL for GFP-His_6_ (0.0026 μmol/cm^2^ or 0.000785 μmol/mg). The order was lysozyme (MW = 14300, pI = 11) > GFP-His_6_ (MW = 28400, pI = 6) > BSA (MW = 69293, pI = 4.7). The adsorption percentage result followed this order. It was found that the molecular size and charge type of the protein had a prominent influence on the adsorption quantity with IMAM. Furthermore, the protein adsorption capacities are much lower than the immobilized copper ion capacity (4.8 μmol/cm^2^). This phenomenon may be attributed to the big protein molecule interacting with numerous copper ions. A large area occupied by the adsorbed protein molecule would block the free copper ions on the surface and reduce the binding of other protein molecules.

As for the fitted values of the equilibrium dissociation constant K_d_, they were: 0.093 ± 0.014 mg/mL for lysozymes, 1.45 ± 0.41 mg/mL for BSA and 0.017 ± 0.004 mg/mL for GFP-His_6_. Since K_d_ is the reciprocal of equilibrium association constant K_a_, it means the order of K_a_ was GFP-His_6_ > lysozyme > BSA. The histidine-tagged protein GFP-His_6_ revealed the strongest affinity with the IMAM, although the lysozyme owned the largest adsorption capacity. It is also worth noting that the K_d_ value of lysozymes for the prepared PAN-based IMAM is smaller than that reported for the poly(vinyl alcohol-co-ethylene)-based IMAM (0.29–0.61 mg/mL) [12], indicating a higher affinity achieved for our IMAM.

The effect due to the charge type of protein was analyzed as follows. According to the pI of protein, the lysozyme was cationic at the adsorption pH condition, whereas BSA and GFP-His_6_ were anionic. Using a pH of 8 for GFP-His_6_ (pI = 6) could make its anionic degree closer to that of BSA (pI = 4.7) at pH 7. In comparison with BSA and GFP-His_6_, the design of tagged histidine had provided a stronger affinity for the green fluorescent protein molecules to bind with the immobilized copper ions of IMAM. Because the specific ligand on IMAM was positively charged copper ions, it should be more difficult for the cationic lysozyme molecules to contact the immobilized copper ions than anionic BSA and GFP-His_6_. However, as analyzed in Section 3.1, a low portion of the COOH groups of membrane were utilized for the formation of specific binding sites. Some COO^−^ groups remained on the membrane surface, providing the nonspecific binding sites with the cationic lysozyme molecules. This could be a reason for the higher lysozyme adsorption capacity.

**Figure 7:**
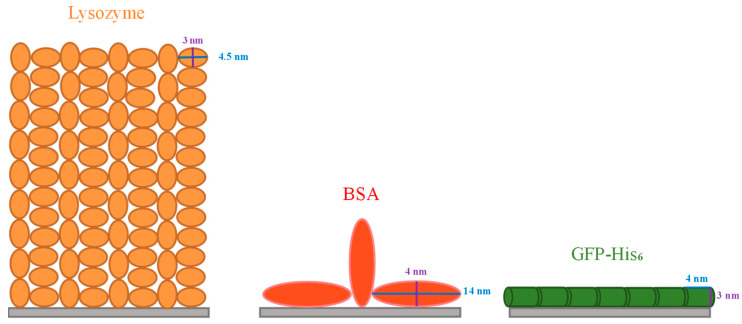
Schematic illustration of possible protein adsorption arrangements on the IMAM.

The maximum biomolecule amount that can be adsorbed on the adsorbent usually relies on its spatial distribution, lateral interaction, conformational change and orientation on the surface [43], which are strongly related to the molecular size and shape. In the present study, it is very likely that the IMAM surface was fully covered with a monolayer of lysozymes, since it is the smallest molecule of the three proteins tested and can bind with the IMAM surface by means of electrostatic interaction and specific binding. The specific surface area for the PAN membrane was 61.8 cm^2^/mg via BET measurement. Through dividing the adsorbed lysozyme quantity of 0.0114 μmol/mg by the specific surface area of the membrane, the surface packing density was 1.11 × 10^14^ molecules/cm^2^. Comparatively, the surface area occupied by each lysozyme molecule is 0.9 nm^2^, which is much smaller than the possible attached area for an ellipsoidal lysozyme molecule. As reported in the literature [43,44,45], the lysozyme is an ellipsoidal molecule with a dimension of 3 nm × 3 nm × 4.5 nm. The surface area attached by a single lysozyme molecule could be either a circle with a diameter of 3 nm (end-on) or an ellipse with a major diameter of 4.5 nm and a minor diameter of 3 nm (side-on). The analysis in the literature [46] indicated that the lysozyme adsorption in a hexagonal packing arrangement corresponds to an adsorbed amount of 3.10 mg/m^2^ for end-on and 2.07 mg/m^2^ for side-on conformation; inversely, the surface area occupied per lysozyme molecule is ca. 8 nm^2^ for end-on and 12 nm^2^ for side-on. Compared to these two values, our result of 0.9 nm^2^ is much smaller than the possible adsorbed area for an ellipsoidal lysozyme molecule in a monolayer arrangement. It implies that the lysozyme adsorption on our IMAM is most likely multilayer, as evidenced by the lysozyme adsorption on a hydrophilic surface in the literature [46]. Based on this presumption, approximately 9 or 13 layers of lysozyme molecules should be bound on our IMAM surface in an end-on or side-on conformation, respectively. Figure 7 shows the schematic illustration for the possible arrangements of lysozyme adsorption in both conformations. A multilayer adsorption arrangement is more likely due to the existence of nonspecific binding sites (COO^−^ groups) for the cationic lysozyme molecules.

From the above interpretation about the adsorption arrangement for lysozymes, it is thus reasonable to conclude that (1) the steric effect existing in the adsorption process was the least for lysozymes, due to them having the smallest molecular size among the tested proteins. (2) Lysozyme molecules might agglomerate into the multimeric protein structure, causing the multilayer binding and a higher adsorption amount. (3) The adsorption amount owing to the electrostatic interaction with IMAM (nonspecific binding with COO^−^ groups) is significant for lysozymes.

The BSA molecule was like an oblate spheroid of 4 nm × 4 nm × 14 nm [47]. After evaluation, the area per BSA molecule adsorbed was about 33 nm^2^, which is between the two molecular orientations: a circle with the diameter of 4 nm (end-on, 12.6 nm^2^) and an ellipse with a major diameter of 14 nm and a minor diameter of 4 nm (side-on, 44 nm^2^). The portion for end-on confirmation might be one-third and that of side-on conformation two-thirds. On the other hand, the GFP-His_6_ molecule was in the shape of a cylinder with a diameter of 3 nm and a length of 4 nm [48]. The surface area occupied for each GFP-His_6_ molecule adsorbed was calculated as 13 nm^2^, suggesting that adsorption took place most likely in the side-on configuration of the GFP-His_6_ molecule (12 nm^2^). Conclusively, both the BSA and GFP-His_6_ adsorption on IMAM should be a monolayer. Based on the above analyses, the schematic illustration of adsorption arrangements for these two proteins is depicted in Figure 7.

#### 3.2.3. Comparison with Literature Data

Table 2 presents a comparison of the immobilized copper ion capacity and protein adsorption capacity obtained for the IMAM prepared in this study with the literature data [12,23,34,38,42,49,50]. Our immobilized copper ion capacity was higher than most of the literature data, even under the use of minor IDA and Cu^2+^ concentrations. It indicates that the preparation process adopted in this study is more environmentally friendly. As for the adsorption capacity, the GFP-His_6_ capacity achieved for our IMAM was greater than that reported in the literature [38], implying that our IMAM should be suitable for special target proteins, such as histidine-tagged proteins. The adsorption capacity of lysozymes was between the literature data, while the BSA adsorption capacity was less than those obtained in the literature [34,50]. The lower BSA adsorption capability might be attributable to the higher portion of side-on conformations in the monolayer adsorption arrangement.

## 4. Conclusions

The PAN-based IMAM was successfully prepared in the present study to capture the target proteins with specific amino acid such as histidine. The optimal and greener reaction conditions were found. The immobilized copper ion capacity was significantly higher than most of the literature data, even under the use of lower chelator and copper ion concentrations. Via the use of the medium imidazole concentration, the recovery of the adsorbed protein on IMAM was greater than 95%. Based on this high desorption efficiency of the prepared IMAM and the advantages of the PAN matrix, resistance to most solvents and the commercial availability, the IMAM in this work was useful in recovering proteins.

Among the three proteins tested in this study, the histidine-tagged protein GFP-His_6_ revealed the strongest affinity with the IMAM. Moreover, the adsorption amount of lysozyme was the highest. The reason was that the geometry of lysozymes was smaller than the other two proteins, leading to the least steric hindrance. In addition, at a neutral pH, lysozymes were positively charged, whereas the pKa of the remaining COOH groups on the IMAM was surpassed, making the IMAM negatively charged. Electrostatic interaction attracted more lysozyme molecules to touch the binding sites on the membrane surface. Other than nonspecific adsorption, the high adsorption amount of the lysozymes may come from the agglomeration of the lysozyme molecules, which enabled the cluster of lysozymes adsorbed to be in a multilayer arrangement. On the contrary, larger BSA and GFP-His_6_ molecules exhibited a monolayer side-on adsorption arrangement in all likelihood.

In comparison with the literature data, the PAN-based IMAM enhanced the adsorption capacity to the histidine-tagged protein GFP-His_6_, making it more suitable and effective for applications involving the separation of target proteins with specific amino acids. In addition, the protein desorption was nearly complete, implying that the reusability and sustainability of the prepared IMAM should be high. Concerning its easy availability and low cost, the PAN-based IMAM is a potential product of an adsorptive membrane. Since the continuous process is more practical in the real-life applications of bioseparation and purification, a future study of the applicability of the PAN-based IMAM should focus on its dynamic performance in an application, such as purification of GFP-His_6_ from *Escherichia coli* or isolation of lysozymes from egg white.

## Figures and Tables

**Table 1:** The results of immobilized copper ions capacity on IMAM under various EDA and EGDGE reaction conditions (based on the use of 0.2 M IDA and 0.05 M CuSO_4_ in the subsequent reactions) via UV-Vis at 800 nm.

EDA Reaction		EGDGE Reaction		Cu^2+^ Capacity (μmol/cm^2^)
Time (h)	Temperature (°C)	Time (h)	Temperature (°C)	
3	50	4	60	3.2 ± 0.04
	60			4.8 ± 0.08
	70			3.5 ± 0.12
2	60	4	60	4.0 ± 0.36
3				4.8 ± 0.08
4				4.6 ± 0.12
3	60	4	40	1.7 ± 0.24
			60	4.8 ± 0.08
			70	3.2 ± 0.24
3	60	2	60	2.4 ± 0.58
		4		4.8 ± 0.08
		6		3.4 ± 0.20

**Table 2:** The comparison of immobilized copper ion capacity and protein adsorption capacity obtained for the IMAM prepared in this study with the literature data.

Membrane Matrix	Chelator	Cu^2+^ Source	Cu^2+^ Capacity	Adsorption pH	Protein	Protein Adsorption Capacity	Ref.
PAN	0.2 M IDA	0.05 M CuSO_4_	4.8 μmol/cm^2^ (253.4 µmol/mL) (1.47 μmol/mg)	pH 7	lysozyme	0.037 μmol/cm^2^ (1.96 μmol/mL) (0.0114 μmol/mg) or 530 μg/cm^2^ (163 μg/mg)	This study
					BSA	0.001 μmol/cm^2^ (0.053 μmol/mL) (0.00031 μmol/mg) or 69.3 μg/cm^2^ (21.5 μg/mg)	
				pH 8	GFP-His_6_	0.0026 μmol/cm^2^ (0.135 μmol/mL) (0.000785 μmol/mg) or 72.7 μg/cm^2^ (22.3 μg/mg)	
Poly(vinyl alcohol- co-ethylene)	0.2 M IDA	0.025 M CuCl_2_	1.13 ± 0.07 μmol/mg	pH 7	lysozyme	199 ± 6 μg/mg	[12]
Hydroxyethyl cellulose-coated nylon	0.75 M IDA	0.01 M CuCl_2_	0.17 μmol/cm^2^	pH 7	lysozyme	321 μg/cm^2^	[23]
Regenerated cellulose	0.2 M IDA	0.1 M CuSO_4_	1.22 μmol/cm^2^	pH 7.4	lysozyme	0.0244 μmol/cm^2^	[34]
					BSA	0.0015 μmol/cm^2^	
Polyvinylidene Fluoride	0.75 M IDA	0.1 M CuSO_4_	0.42–0.53 μmol/cm^2^	pH 7	lysozyme	0.055–0.085 μmol/cm^2^	[42]
Surface-modified polyethylene hollow fiber	1 M IDA	0.5 M CuSO_4_	1500 μmol/mL	pH 7	lysozyme	1–8.5 μmol/mL	[49]
Glycidyl methacrylate-grafted polyethylene hollow fiber	0.425 M IDA	0.01 M CuSO_4_	180 µmol/mL	pH 8	BSA	0.26 µmol/mL	[50]
Polyethersulfone	2 M IDA	0.5 M CuSO_4_	(5.82 ± 0.22)×10^−3^ μmol/mg	pH 8	GFP-His_6_	1.14–3.01 μg/mg	[38]

## Data Availability

Not applicable.
